# Imaging Evaluation of Platelet-Rich Fibrin in Post-Exodontic Bone Regeneration: A Systematic Review

**DOI:** 10.3390/dj11120277

**Published:** 2023-11-29

**Authors:** Magdalena Molina-Barahona, Bolívar Delgado-Gaete, Denia Morales-Navarro, Joaquín Urbizo-Vélez, Renata Avecillas-Rodas

**Affiliations:** 1 Maxillofacial Radiology Department, Faculty of Dentistry, Universidad Catolica de Cuenca, Cuenca 010101, Ecuador; bolivar.delgado@ucacue.edu.ec; 2 Faculty of Dentistry, Universidad de Ciencias Medicas de la Habana, Havana 104000, Cuba; 3 Maxillofacial Surgery Department, Universidad de Ciencias Medicas de la Habana, Havana 104000, Cuba; deniamorales@infomed.sld.cu (D.M.-N.); joaquin.urbizo@infomed.sld.cu (J.U.-V.); 4 Faculty of Dentistry, Catholic University of Cuenca, Cuenca 010101, Ecuador; renata.avecillas@est.ucacue.edu.ec

**Keywords:** platelet-rich fibrin, bone regeneration, dental extraction, physiological healing, imaging evaluation

## Abstract

Tooth extraction is the most common procedure in dental practice. However, in the long term, it may cause alveolar ridge atrophy. This systematic review aimed to evaluate the role of platelet-rich fibrin (PRF) in post-exodontic alveolar ridge preservation in terms of its effectiveness in the regeneration of bone tissue as assessed by imaging and its efficacy compared to physiological bone healing. The study is presented in accordance with the preferred reporting items for systematic reviews and meta-analyses (PRISMA) guidelines. This systematic review was conducted using electronic databases such as PubMed, Scopus, Web of Science, and Science Direct. The gray literature search was conducted in the New York Academy of Medicine Grey Literature Report. All the studies in this systematic review were randomized controlled trials (RCTs). The risk of bias was performed according to the Cochrane Handbook for Systematic Reviews of Interventions 6.2 (RevMan 6.2). Considering the inclusion and exclusion criteria, we included 17 randomized clinical trials published up to 2022 investigating the efficacy of PRF in post-exodontic bone regeneration. Based on the results of clinical studies, it can be stated that despite not being statistically significant, PRF promotes neoformation and prevents bone loss between three and four months post-extraction.

## 1. Introduction

Tooth extraction is the most common procedure in dental practice. However, in the long term, it may cause alveolar ridge atrophy, leading to postoperative complications. Healing of soft and hard tissues is a complex biological process that results in the restoration of the affected tissue, requiring platelets to release autologous growth factors [1]. However, tooth extraction causes a dimensional loss in the height and width of the alveolus during physiological bone regeneration as part of the healing process [2]. Between three and six months post-extraction, the bone width is reduced by approximately 2.6–4.6 mm and the height by 0.4–3.9 mm [3]. Being a rapid and continuous process, it results in long-term atrophy of the alveolar border, causing a 50–60% loss of the entire alveolar ridge in the first three months [4].

The alveolar ridge can be preserved within 3–6 months, depending on the morphology of the defect and the biomaterial applied. Autologous materials are easy to prepare, regulate inflammation, and support proper postoperative healing of soft and hard tissues [5].

Platelet concentrates are compounds obtained directly from blood and comprise leukocytes, platelets, plasma proteins, and growth factors that are concentrated by different centrifugation processes [6]. In 1974, Ross et al. first identified a growth factor immersed in the platelets of an autologous fibrin matrix responsible for the mitogenic response in the bone periosteum during the average healing period. This process involves blood centrifugation [1,4]. 

The first generation of platelet concentrates is platelet-rich plasma (PRP), described by Marx et al. in 1998. PRP mainly includes platelets, and leukocytes are eliminated during its preparation [7]. The process requires two centrifugation steps and adhesion substances such as thrombin, an anticoagulant, or calcium chloride, which may produce antibodies, such as factors 6 and 9, posing a life-threatening risk to patients with coagulopathies [8,9]. 

Plasma rich in growth factors represents another derivative of platelet concentrates; however, similar to PRP, it requires a relatively high centrifugal force during preparation and can trigger a long-term decrease in angiogenesis [10].

The second generation of platelet concentrates is platelet-rich fibrin (PRF), first reported by Choukron et al. Its preparation requires only 10 mL of autologous blood. Similar to that of other platelet concentrates, PRF preparation is by centrifugation but varies in that only one step is involved and does not require anticoagulants or bovine thrombin [11]. Thus, it contains high levels of growth factors slowly released within 7–14 days after its use [12,13,14,15,16,17,18,19].

Several systematic reviews have attempted to consolidate all available evidence on PRF use. However, they mainly focused on the clinical findings and did not evaluate alveolar preservation through two- or three-dimensional imaging [3,5,20]. Two-dimensional imaging has been used to validate bone regeneration but may mask the information that can only be observed through three-dimensional imaging [13,17,20,21,22]. Cone-beam computed tomography (CBCT) is regarded as the optimal technique for acquiring three-dimensional images in axial, sagittal, and coronal planes. This imaging process allows clear visualization of various aspects, including the width and height of the alveolar ridge. It is worth mentioning that one of the key advantages of CBCT is its utilization of Hounsfield units (HU), which aid in the assessment of neoformed bone tissue quality. Consequently, this feature can greatly facilitate the qualitative identification of bone regeneration [13,20]. This study evaluated the role of PRF in post-exodontic alveolar ridge preservation regarding its effectiveness in bone tissue regeneration as assessed by imaging and its efficacy compared to physiological bone healing.

## 2. Materials and Methods

This systematic review was reported according to the PRISMA 2020 guidelines for reporting systematic reviews [23]. The review protocol has been published in PROSPERO (Ref. No: CRD42022332992).

### 2.1. Inclusion and Exclusion Criteria 

Original research articles that reported treatment outcomes of PRF versus spontaneous healing (blood clots) or biomaterials in post-exodontic cases were selected for inclusion in the current review. The population (P) studied comprised patients over 18 years of age who underwent dental extractions. The interventions (I) included were the use of PRF in post-exodontic sockets. The comparison (C) was made with the natural healing process of post-exodontic sockets. The primary outcomes (O) were focused on imaging assessments of bone regeneration, with a minimum follow-up duration of two to three months. Animal studies were excluded. The treatment with PRF combined with biomaterials of different origins was excluded. Only randomized controlled trials (RCTs) in humans in a split-mouth or parallel design with reasonable controls and at least 10 patients were included (Table 1).

### 2.2. Types of Included Studies

Randomized controlled trials (RCTs) in humans in a split-mouth or parallel design with reasonable controls and at least 10 patients were included. The publications included were written in English, Spanish, or Portuguese. Whenever missing information was encountered, the authors of that paper were contacted and requested to provide further information. The volume of information was substantial, and the randomized controlled trials (RCTs) positioned at the apex of the evidence hierarchy are acknowledged to possess the least degree of bias. Consequently, we selected this specific experimental design with the aim of obtaining more dependable and conclusive findings.

### 2.3. Search Strategy 

This review was based on the PICO strategy [24] and a bibliographical search. The electronic search was performed in the following registries for papers published up to April 2022: PubMed, Scopus, Web of Science, and Science Direct. The gray literature search was conducted in the New York Academy of Medicine Grey Literature Report. For the database searches, keywords and Boolean operators specific to each database were used in the search strategy (the detailed search strategy is presented in Table 2). The titles and abstracts of all articles retrieved in the database searches were reviewed independently by three reviewers (M.M.-B., B.D.-G., and J.U.-V.). The full-length texts for the selected studies were then retrieved, and a final list of studies was compiled based on the criteria mentioned above. Any disagreements were noted and resolved in a discussion between the three reviewers, and four additional reviewers were available for consultation when necessary. Studies excluded after obtaining the full text were documented separately, along with the relevant reasons for exclusion.

### 2.4. Data Extraction and Quality Assessment 

**Figure 1:**
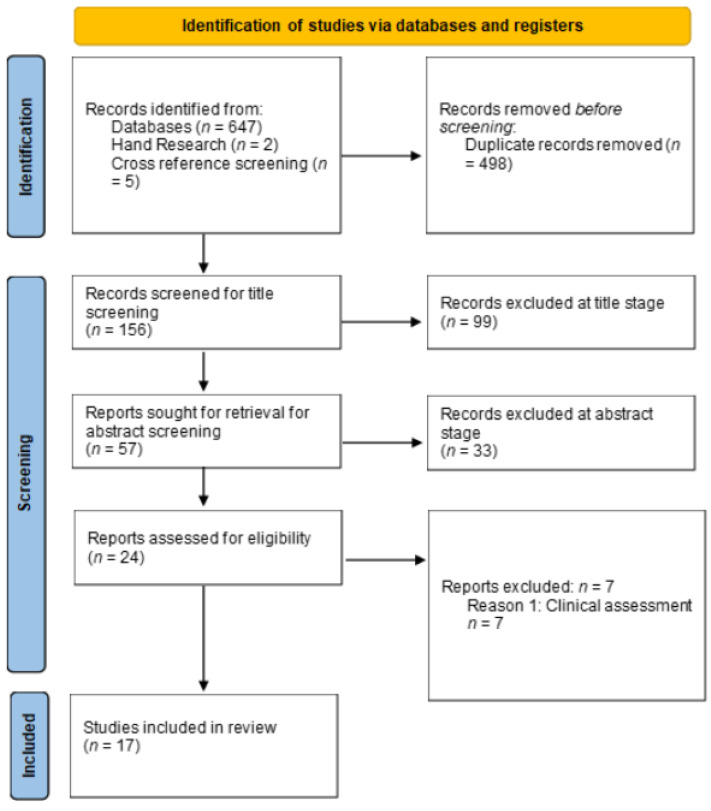
PRISMA 2020 flow diagram for RCTs which included searches of databases and registers only.

Data were extracted from the included studies after the full-text screening. The data representing the outcomes of imaging evaluation of bone regeneration, dimensional changes (width and height), and quality of the neoformed bone tissue were summarized using a narrative review. The quality of the selected RCTs was reviewed to assess bias risk. The assessment was performed according to the Cochrane Handbook for Systematic Reviews of Interventions 6.2 (RevMan 6.2), which categorizes study evidence as “low,” “high,” and “unclear.” The following categories were analyzed: randomization, concealed allocation, blinding of participants and specialists, blinding of outcome assessment, and RCT abandonment [25]. Four independent investigators (M.M.-B., B.D.-G., D.M.-N., and J.U.-V.) performed the evaluations mentioned above based on the full-text articles. Disagreements were resolved through guided discussion between the four authors. The selection flowchart of primary studies with bias for the 17 included articles is shown in Figure 1 and describes the global evaluation of the risk of bias and evaluation of the risk of bias by domain using the Cochrane tool.

## 3. Results

### 3.1. Details of the Included Studies

The details of the search results are presented in the PRISMA flowchart shown in Figure 1. A total of 654 articles were identified from the databases (Web of Science: 76, PubMed: 82, Scopus: 19, Science Direct: 16, gray literature: 461). In total, 17 were included in the qualitative analysis (Figure 1). The characteristics of the included studies are described in Table 3, including the study design, number of patients, age range, number of pieces that involved the use of PRF and control pieces, control time, and reason for tooth extraction. Table 4 shows the variables used and the evaluation method of each study, whereas Table 5 summarizes the results using the following criteria: alveolar bone loss with PRF and in the control, and presence of statistical significance and effect of platelet concentrate reported in the study. In addition, the analysis showed a high degree of heterogeneity among the randomized clinical trials, and, therefore, conducting a meta-analysis on these variables was not feasible.

### 3.2. Description of the Included Interventional Studies 

This review analyzed 17 parallel RCTs. Thirteen studies compared PRF treatment alone with physiological healing or blood clotting without additional treatment [1,2,3,5,13,14,17,19,20,22,26,27,28,29,30]. One study included three groups and compared the use of PRF and PRF with mucosal flap with spontaneous healing [21]. One study included three groups and compared advanced PRF (A-PRF) treatment, A-PRF + freeze-dried bone allograft (FDBA), FDBA alone, and spontaneous healing [26]. One study compared the use of leukocyte- and platelet-rich fibrin (L-PRF) or platelet-rich fibrin (A-PRF) with spontaneous healing [10]. Finally, one study compared the use of PRP, PRF, hydroxyapatite, and spontaneous healing [30].

Patients were mainly studied with or without requiring dental exodontia (including the third molar) and/or implants were required for some but not all of the patients included in the studies [1,3,5,10,13,14,17,19,20,21,22,26,27,28,29,30]. Additionally, some studies focused only on uniradicular teeth or premolars [2,10,21,26] (Table 3). Most studies did not report or specify the treated socket and/or ridge morphology. All studies reported the presence of 50% buccal and/or lingual bone walls [1,3,5,10,13,14,17,19,20,21,22,26,27,28,29,30]. Most studies reported traumatic exodontia [1,2,3,5,10,13,17,19,20,21,22,26,27,28,29,30].

### 3.3. Quality Assessment of the Included Studies 

**Figure 2:**
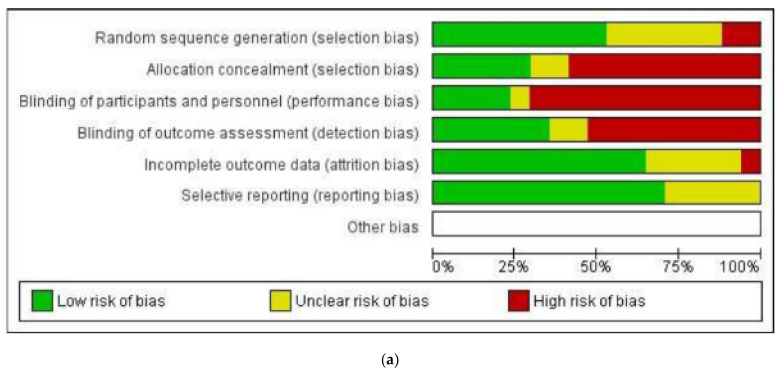

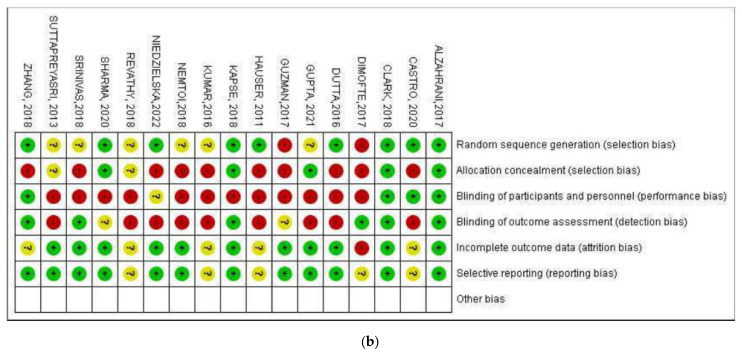
Cochrane analysis: Quality assessment of the studies included in the analysis: (**a**) summary of the risk of bias assessment; (**b**) detailed assessment discriminated by risk of bias criterion and by individual study [1,2,3,5,10,13,14,17,19,20,21,22,26,27,28,29,30].

According to our criteria on the risk of bias in RCTs [25], the highest risk of bias was observed in “participant and professional blinding” and “allocation concealment” (Figure 2). A potential explanation is that patients and professionals were aware of the experimental method being executed.

## 4. Discussion

All the studies reported only the number of revolutions per minute (rpm) and centrifugation time but did not refer to the centrifuge design and detail the relative centrifugal force applied in the procedures [1,3,5,10,13,14,17,19,20,21,22,26,27,28,29,30].

Seventeen studies evaluated the dimensional changes and bone regeneration using different methods of analysis. 

Clark et al. studied a clinical measurement method using metal devices to corroborate bone loss 6 months post-extraction [26]. They also analyzed the changes in bone dimensions 15 weeks later using an alginate impression, a periodontal probe, and X-ray analysis. The results showed that the PRF group had a significantly smaller reduction in ridge height than the control group; however, there was no statistically significant difference in alveolar bone width or bone tissue quality. In contrast, Hauser et al. reported a significantly lower percentage of alveolar ridge width resorption in the PRF group than in the control group after eight weeks [21].

Dimensional alveolar atrophy was previously measured using cone-beam computed tomography (CBCT) at three months post-exodontia in a study by Castro et al. [10]. The results did not show statistical significance regarding PRF use compared with a control site in terms of the width and height of the buccal and palatal/lingual tables. However, Niedzielska et al. showed that PRF use at six months significantly reduced bone loss in the width and height of the alveolar process [28].

In contrast, Nemtoi et al. performed the evaluation six months post-extraction and showed a statistically significant bone loss in the control group, where more accelerated filling of the alveolus was demonstrated [19]. However, Dimofte et al. reported that although no significant differences were found in bone loss in terms of width and height, a relationship with physiological healing was noticed [30]. In addition, PRF was shown to prevent advanced bone loss in the alveoli of uniradicular and multiradicular teeth over time. Finally, Srinivas et al. evaluated bone height at 3 months post-exodontia in cases of PRF use and observed increased alveolar ridge in the control group, establishing that PRF is ideal for bone regeneration [14].

Post-exodontic bone density was assessed using the grayscale known as Hounsfield units (HU) [9]. Srinivas et al. evaluated bone density using HU in the apical and middle thirds of the alveolus with PRF and found that it was higher than that in the control group at three months [14]. However, Niedzielska et al. found no significant statistical difference [28]. Finally, the study by Nemtoi et al. showed the bone quality formed at six months, with the blood clot, with clinical significance compared with the intervention group [19]. Guzmán et al. analyzed the quality of bone tissue (HU) formed using PRF. They reported that the quality of the uniradicular and multirooted teeth was significantly higher at 60 days in the control group (physiological healing) [29]. PRF application to other materials (e.g., L-PRF) showed that despite the absence of significant differences, the presence of greater bone density is relevant when analyzing the qualitative aspects of the neoformed bone tissue [10].

The evaluation results of alveolar preservation using two-dimensional techniques were reported by Revathy et al., who used orthopantomography as the evaluation method [1]. The analysis was performed at one, three, and six months post-exodontia and showed statistically significant differences between PRF and physiological healing, thus concluding that PRF allows the maintenance of the alveolar ridge [1]. In contrast, Sharma et al. demonstrated no statistical significance at 16 weeks of evaluation; however, they showed that bone tissue neoformation was accelerated [22].

Sharma et al. found that although the quality of the bone tissue was higher, it was not significantly different from the traditional procedure [22]. In contrast, Suttapreyasri and Leepong evaluated bone loss on periapical radiographs using the parallelism technique at 8 weeks [2]. The height was not significantly different in the control group. Hauser et al. evaluated mesial and distal measurements at 8 weeks and observed statistical significance in both cases [21].

Zhang et al. monitored bone loss for three months in the lingual vestibular table and the width of the alveolar bone but did not observe statistically significant differences with the control group [3]. Nevertheless, they concluded that PRF is advantageous for preserving the alveolar ridge. Sharma et al. evaluated bone regeneration of the lamina dura at 8 and 16 weeks and obtained statistically significant values for bone regeneration at 16 weeks [22].

Gupta et al. studied the percentage of bone loss at one, three, and six months post-extraction [20]. The values were statistically significant in the control group, and the authors concluded that the use of PRF is valid for bone regeneration of the post-exodontic alveolus. Alzahrani et al. used periapical radiography to evaluate the loss of alveolar ridge width after one, four, and eight weeks [13]. They found statistically significant values in the first and fourth weeks but not in the eighth week; however, the efficiency of cell regeneration and osteoblastic activity increased, contributing to bone loss reduction. Another study that evaluated the regeneration of the lamina dura was that of Dutta et al., who determined that PRF use led to a significantly lower proportion of bone loss at six months [27].

The bone tissue quality was also evaluated using periapical radiographs using the same principle as grayscale orthopantomography. Kumar et al. analyzed bone fill at six months but did not observe statistically significant differences [5]. Kapse et al. studied bone density and trabecular patterns for 16 weeks and found that the quality of the bone tissue treated with PRF was significantly higher compared to that in the control group [17].

In contrast, Alzahrani et al. observed at eight weeks a great capacity for bone tissue formation compared to physiological healing, which increases cellular proliferation of the experimental alveolus [13]. Similarly, Niedzielska et al. found no significant differences in bone density between the PRF and control groups [28]. Finally, the quality of the trabecular pattern was evaluated by Dutta et al., who corroborated that in the first, second, and sixth months, it was greater in the PRF group than in the control group [27].

Clark et al. and Hauser et al. studied core biopsies using micro-CT [21,26]. After eight weeks, bone and tissue volume analyses showed no differences between the PRF and control groups. Bone density measurements at 15 weeks showed no significant differences either. Castro et al. reported a significantly higher percentage of bone volume when comparing the PRF group with the control group [10].

Platelet concentrates, especially PRF, have been increasingly used in regenerative stomatology [1]. PRF is used to improve wound healing and bone regeneration [21]. Several RCTs have recently evaluated the clinical evidence on the role of PRF in wound healing [2,3,5,13,14,17,19,22,26,27,28,29,30]. Most of them analyzed more than one indication and used various inclusion criteria, making it difficult to determine specific indications [3,13,14,22,28,29]. In addition, the objective of analyzing bone regeneration is usually long-term implant placement, but little is known regarding bone regeneration in the vestibular and lingual/palatal bone tables. PRF can stimulate bone regeneration in situ without waiting for a normal body response. Furthermore, owing to the minimal number of cytokines in PRF, the bone regeneration effect is limited, and the shape of the post-exodontic alveolar ridge cannot be maintained at eight weeks [21].

Platelet factors provide satisfactory outcomes in the postoperative period and for future implant placement through proper bone regeneration, which prevents alveolar atrophy typical of dental exodontia [4]. Guzmán et al. argued that the literature does not present coherent control groups, and additional related factors have been added [29]. 

Previous studies have compared PRF with mineralized bone-substitute materials (e.g., FDBA) [26]. PRF is an autologous bioactive blood concentrate based on blood elements, including platelets and leukocytes embedded in a fibrin network; thus, it does not present the physicochemical characteristics of conventional biomaterials and, therefore, is not comparable with other bone substitutes or collagen membranes. This shows the importance of evaluating PRF compared with materials of similar characteristics for bone regeneration [4].

This systematic review defined natural blood clots as a variable of analysis in spontaneous wound healing to evaluate the regenerative effect and the qualitative and quantitative efficacy of PRF. Therefore, the following focused question was posed: In post-exodontic patients, what is the efficacy of PRF in bone regeneration compared to the untreated sockets (blood clots) assessed by imaging?

A literature search identified only 17 RCTs that examined the effects of PRF on spontaneous bone healing. The bias risk was relatively high in most studies, especially for patient blinding and outcomes [1,2,3,5,10,14,17,19,20,21,22,26,27,28,30].

Furthermore, no study has mentioned the anatomy of the exodontic area and the quality and dimensions of the vestibular and lingual/palatal post-exodontic walls and analyzed marginal bone resorption. These factors are important for quantitatively evaluating bone regeneration and future alveolar atrophy; therefore, obtaining better-quality data in future studies is vital.

Additionally, when analyzing PRF, knowing the preparation protocol is important because it is a blood derivative prepared during the surgical procedure for each patient, and errors may occur during its preparation [18]. In this regard, several centrifugation protocols have been reported. Still, the parameters do not include the centrifugation force applied, which has a crucial influence on PRF bioactivity and, therefore, therapeutic efficacy. Investigations have affirmed that the application of high centrifugation forces in PRF preparation results in a lower proportion of platelets, leukocytes, and growth factors than that of a low centrifugation speed [26]. Studies define this low centrifugation force as the ideal parameter in PRF preparation; however, other indispensable components of the procedure can be mentioned such as the speed, which is usually adjustable; the centrifugal force (rpm), which is not visible in the centrifuge but can be calculated according to the radius and centrifugation time; and the centrifugation time, which affects the quality of the PRF preparation [4].

Most of the studies describe only the applied rpm but not the centrifugal radius or the resulting centrifugal force [2,3,5,10,13,14,17,19,20,21,22,26,27,28,29,30]. Only one study described rpm and used the first protocol introduced by Choukrouns et al. for PRF preparation with a relatively high speed (2700–3000 rpm for 10–12 min) [1]. In addition, two studies used different PRF protocols to prepare a more advanced compound using 1300 rpm [10,26]. Applying different preparation protocols results in different compound qualities, which may interfere with clinical outcomes [31]. Therefore, a preparation guide for platelet compounds based on scientific data obtained from different investigations is necessary.

According to the data analyzed, the role of PRF in bone regeneration demonstrated different results depending on the time of evaluation and methodology applied. Several studies have reported bone neoformation after 8 weeks [2,3,10,13,14,17,21,22,26,29,30]. Only six studies evaluated bone regeneration at 24 weeks [1,5,19,20,27,28].

Similarly, four investigations demonstrated significantly lower bone resorption in the PRF group than in the control group, especially considering the vestibular bone table [3,13,21,27]. Additionally, the quality of the bone tissue (HU) formed in the alveolus showed less resorption in the PRF group than in the control group (physiological healing) between 8 and 24 weeks, and the measurement between 1 and 3 mm caudal to the alveolar ridge was maintained under inadequate conditions in the PRF group [17,30].

Despite the limitations of the data obtained, this review highlights that PRF accelerates bone regeneration during the early healing phase [1,3,10,20,26]. It has also been shown that PRF is effective because it prevents post-exodontic alveolar atrophy; however, it cannot prevent long-term atrophy because its effectiveness decreases after six months [3,10,19,20,21,26,27].

These findings are estimable if PRF is considered a bioactive and autologous fibrin scaffold that is different from prefabricated biomaterials based on collagen or bone substitutes [31]. One study showed that the action of PRF decreases after 2–3 weeks, a period considered sufficient to exert its effect on early healing [20]. In contrast, the activity of conventional biomaterials, such as collagen matrices or bone substitutes, may vary from three months to a few years, depending on the characteristics of the composite [30]. Therefore, PRF should be considered a fibrin scaffold, not a classic biomaterial [31], and be used as an adjuvant therapy to other biomaterials for bone regeneration (e.g., xenogeneic, allogeneic, and synthetic).

Considering the preparation protocols of PRF, the clinical application is quite wide and it should not be considered a classical biomaterial in guided bone regeneration and guided tissue regeneration processes [31,32]. In guided tissue regeneration, the materials used are acellular and inactive and need a certain amount of time to integrate into the surgical bed, thus allowing cell migration and bone regeneration [27]. PRF is a bioactive scaffold that includes the platelets necessary for bone regeneration, accelerates the phases of wound healing, and initiates the bone regeneration process earlier than in the case of classic biomaterials [4].

Physiological post-exodontic atrophy is a rapid and continuous process in which the first three months are crucial for bone loss in approximately 50–70% of cases, which makes the use of a material such as PRF necessary to prevent alveolar atrophy [9,18,19,20,21,22,23,24,25,26,27,28,29,30,31]. However, after six months, the effect of PRF begins to diminish, and bone is generated in a lower proportion [20]. Concluding on the efficacy of PRF is not feasible. Therefore, evaluating more RCTs with suitable parameters for their execution is necessary.

None of the investigations that include cone-beam (CBCT) mentioned the rotation type used (180° or 360°), size of the field of view, amount of radiation emitted, or time required to obtain the CBCT images applied in the experimental group. These data are vital because doubt could arise regarding whether this imaging test, without any specification, generates alterations in the mitotic process of the newly formed bone tissue, which could reduce bone regeneration efficacy in the long term. This parameter could be considered the cause of alveolar atrophy at six months.

In summary, the analysis of available scientific evidence from 17 randomized RCTs highlights the efficacy of PRF in post-exodontic bone regeneration. Thus, despite not being statistically significant, PRF promotes new bone formation and prevents bone loss between three and four months, consistent with the protocols that use high centrifugation forces. However, the number of studies in this field is relatively limited. Therefore, the risk of bias was high.

This review has some limitations. First, the methodologies employed for the handling of PRF varied, including centrifuge revolutions, centrifuge models, and blood collection tubes. Additionally, diverse approaches to imaging evaluation were used. Furthermore, the assessment of the risk of bias revealed that several of the studies provided a relatively low evidence level. The included studies also used disparate combinations of preparation procurement and surgical techniques. Owing to the heterogeneity of the RCTs, conducting a meta-analysis on these variables was not feasible. Therefore, a randomized clinical trial with a greater emphasis on standardizing variables is necessary. This approach could yield more accurate and reliable insights into the clinical effects of PRF in alveolar preservation. The exclusive dependence on randomized controlled trials (RCTs) imposes limitations on the comprehensive qualitative assessment of the subject matter; hence, it is imperative to exercise caution when analyzing the offered material.

## 5. Conclusions

This review focused on imaging evaluation of the efficacy of PRF in post-exodontic bone regeneration compared with physiological healing (blood clot). Despite the limitations of the data obtained, it can be concluded that PRF can improve alveolar ridge preservation. Future clinical studies should compare various PRF preparations and application protocols to gauge their simplicity of use and determine any significant differences these preparations and protocols may have on clinical outcomes.

Although the review focused on RCTs, a relatively high risk of bias was identified, especially in two aspects: blinding of the outcome assessment and blinding of participants and staff. This situation can be justified by the type of experiment performed, as both the patient and professional need to be aware of it. In addition, since the analyzed information showed a high degree of heterogeneity among the identified RCTs, conducting a meta-analysis on these variables was not feasible.

## Figures and Tables

**Table 1:** Inclusion and exclusion criteria.

Inclusion	Exclusion
Patients age: 15–99 years	Animal studies
Treatment with PRF versus spontaneous healing (blood clots) or biomaterials, that is, bone-substitute materials, collagen membranes, and any other membrane of different origin	Treatment with PRF combined with biomaterials of different origins
Post-exodontic alveolus	Patients with periodontal or bone defects, including dehiscence or fenestrations
Treatments without any additional chemical or physical agents in the post-exodontic socket, except the use of suture materials	Patients with immediate implant placement
Patients not taking anticoagulants	Studies not reporting imaging data or unrelated to PRF

**Table 2:** Electronic databases and research strategies.

Pubmed P—I #1((((“Tooth Extraction/adverse effects”[Mesh] OR “Tooth Extraction/classification”[Mesh] OR “Tooth Extraction/methods”[Mesh]) OR Tooth Extractions OR Extractions, Tooth OR Extraction, Tooth) AND ((“Platelet-Rich Fibrin/cytology”[Mesh] OR “Platelet-Rich Fibrin/diagnostic imaging”[Mesh] OR “Platelet-Rich Fibrin/immunology”[Mesh] OR “Platelet-Rich Fibrin/physiology”[Mesh] OR “Platelet-Rich Fibrin/radiation effects”[Mesh]) OR Platelet Rich Fibrin OR Fibrin, Platelet-Rich)) AND C # 2((“Wound Healing”[Mesh]) AND (“[Mesh] OR “Wound Healing/immunology”[Mesh] OR “Wound Healing/radiation effects”[Mesh])OR Healing, Wound OR Healings, Wound OR Wound Healings)) AND O # 3 (“Radiologic evaluation/physiology” [Mesh] OR Regenerations, Bone OR Regeneration, Bone OR Bone Regenerations OR “Cone-Beam Computed Tomography” [Mesh] OR” (CBCT)) #1 AND #2 AND #3
Scopus P-I #1 (TITLE-ABS-KEY (tooth extraction) OR TITLE-ABS-KEY (Extraction, Tooth)OR TITLE-ABS-KEY (Platelet-Rich Fibrin) OR TITLE-ABS-KEY (PRF)) C #2 (TITLE-ABS-KEY (Physiological healing) OR TITLE-ABS-KEY (Blood clot)OR TITLE-ABS-KEY (Wound Healing)) O #3 ((TITLE-ABS-KEY (bone regeneration) OR TITLE-ABS-KEY (Radiologic evaluation) OR TITLE-ABS-KEY(Cone beam computed tomography) OR TITLE-ABS-KEY (CBCT)) #1 AND #2 AND #3
Science Direct P—I #1 Tooth Extraction, Platelet-Rich Plasma C # 2 Physiological healing, Blood clot O # 3 Bone Regeneration, Cone-Beam, Computed Tomography #1 AND #2 AND #3
Web of Science (Core Collection) P—I #1 TS = (Tooth Extraction OR Extraction tooth OR Platelet-Rich Fibrin OR PRF) Indexes = SCI-EXPANDED, SSCI, A&HCI, CPCI-S, CPCI-SSH, ESCI, CCR-EXPANDED, IC Timespan = All years C #2 TS = (Wound Healing OR Physiological healing OR Blood Clot) Indexes = SCI EXPANDED, SSCI, A&HCI, CPCI-S, CPCI-SSH, ESCI, CCR-EXPANDED, IC Timespan = All years O #3 TS = (Bone regeneration OR) Indexes = SCI-EXPANDED, SSCI, A&HCI, CPCI-S, CPCI-SSH, ESCI, CCR-EXPANDED, IC Timespan = All years #1 AND #2 AND #3
New York Academy of Medicine Grey Literature Tooth Extraction, Platelet-Rich Fibrin, Wound Healing, Physiological Healing, Bone regeneration, Radiologic evaluation, Cone Beam.

Abbreviations: PICO Strategy: P: Population; Intervention: Comparator; O: Outcome.

**Table 3:** Data summary of the study’s enhancement from the RCTs.

First Author/Country, Geographic Region	Study Design	Sample Size	Age Range	Number of Patients		Intervention		Control Time	Reason for the Extraction
				Test	Control	Type Test	Control		
Clark et al., 2018, United States, North America [26]	RCT	40	Median age 58 years	10	10	PRF	A-PRF + FDBA; FDBA; Blood clot	15 weeks	Uniradicular pieces in need of replacement with dental implants
Revathy et al., 2018, India, Asia [1]	RCT	25	Between 18 and 35 years	25	25	PRF	Blood clot	4, 12, and 24 weeks	Impacted mandibular third molar
Castro et al., 2018, Belgium, Europe [10]	RCT	21	Over 18 years	30	30	PRF	L-PRF; Blood clot	12 weeks	Non-treatable uniradicular pieces located in esthetic areas
Suttapreyasri and Leepong, 2013, Thailand, Asia [2]	RCT	8	Between 22 and 44 years	10	10	PRF	Blood clot	1, 2, 4, 6, and 8 weeks	Symmetrical extraction premolars
Hauser et al., 2011, Switzerland, Europe [21]	RCT	23	Between 22 and 75 years	9 PRF–6 PRF—FLAP	8	PRF	Blood clot; PRF FLAP	1, 2, 5, and 8 weeks	Premolars for implant replacement due to: Endodontic treatment failures, root fractures, advanced carious lesions and periodontal compromise
Sharma et al., 2020, India, Asia [22]	RCT	30	Between 18 and 45 years	30	30	PRF	Blood clot	3, 7, and 24 days and 12 weeks	Bilateral exodontia of mandibular first or second molars
Kumar et al., 2016, India, Asia [5]	RCT	34	Between 18 and 40 years	34	34	PRF	Blood clot	2, 4, and 6 months	Impacted mandibular third molar
Zhang et al., 2018, China, Asia [3]	RCT	28	No details on age	14	14	PRF	Blood clot	1, 16, and 48 weeks	Fractured teeth or root remnants
Kapse et al., 2018, India, Asia [17]	RCT	30	Between 18 and 40 years	30	30	PRF	Blood clot	1, 4, 7, and 14 days; and 8 y 16 weeks	Bilateral impacted third molars
Gupta and Agarwal, 2021, UK, Europe [20]	RCT	20	Between 18 and 35 years	20	20	PRF	Blood clot	1, 3 days; and 1, 4, and 24 weeks	Bilateral impacted third molars
Alzahrani et al., 2017, Saudi Arabia, Asia [13]	RCT	24	Between 25 and 50 years	12	12	PRF	Blood clot	1, 4, and 8 weeks	Exodontia of a tooth due to root fracture, poor periodontal prognosis, failure of endodontic treatment, advanced caries
Srinivas et al., 2018, India, Asia [14]	RCT	30	Between 20 and 50 years	30	30	PRF	Blood clot	24 h and 3 months	Upper or lower teeth with/without chronic periodontal disease
Dutta et al., 2016, India, Asia [27]	RCT	40	Between 17 and 36 years	40	10	PRF	Blood clot; PRF + HA	1, 2, and 6 months	Extraction of mandibular third molars
Niedzielska et al., 2022, Poland, Europe [28]	RCT	50	No details on age	48	41	PRF	Blood clot	Immediate postoperative and 6 months	Exodontia of 2 homonymous maxillary or mandibular teeth: endodontic failure, coronary fracture
Nemtoi et al., 2018, Romania, Europe [19]	RCT	40	Between 12 and 20 years	20	20	PRF	Blood clot	Immediate postoperative and 6 months	Exodontia of upper or lower teeth
Guzmán et al., 2017, Ecuador, South America [29]	RCT	30	Between 16 and 27 years	30	30	PRF	Blood clot	60 days	Extraction of mandibular third molars
Dimofte et al., 2017, Romania, Europe [30]	RCT	63	Between 18 and 58 years	No details	No details	PRF	Blood clot	7 days; and 12 weeks	Bilateral extraction, presence of retained roots, non-restorable caries

Abbreviations: A-PRF: advanced PRF; FDBA: freeze-dried bone allograft; PRF: platelet-rich fibrin; L-PRF: leukocyte- and platelet-rich fibrin; HA: hydroxyapatite; RCT: randomized controlled trial.

**Table 4:** Summary of the variables and evaluation methods in the RCTs included in the analysis.

Author	Variables	Evaluation Method
Clark et al. [26]	Loss of ridge height Loss of ridge width (coronal) Loss of ridge width (central) Loss of ridge width (apical)	Radiographic evaluation (micro-CT) Histomorphometric evaluation
Revathy et al. [1]	Bone healing (osteoblastic activity)	Radiographic evaluation: Panoramic X-ray
Castro et al. [10]	Change in horizontal ridge level of 1 mm Change in horizontal ridge level between −3 and −5 mm vertical bone resorption in the vestibular and palatal table	Radiographic evaluation: (CBCT)
Suttapreyasri and Leepong [2]	Bone resorption of the alveolar ridge Soft tissue healing	Clinical evaluation Radiographic evaluation
Hauser et al. [21]	Bone tissue healing Soft tissue healing	Clinical evaluation Radiographic evaluation (periapical parallelism technique) Histological evaluation (micro-CT)
Sharma et al. [22]	Bone tissue healing Soft tissue healing	Clinical evaluation Radiographic evaluation: panoramic X-ray
Kumar et al. [5]	Bone tissue healing Healing of soft tissues Pain	Clinical evaluation Radiographic evaluation: periapical
Zhang et al. [3]	Changes in alveolar ridge height, width, bone density Bone density	Clinical evaluation Radiographic evaluation Histological evaluation
Kapse et al. [17]	Bone regeneration (lamina dura, bone density, and trabecular pattern). Pain Edema	Clinical evaluation (VAS, edematization) Radiographic evaluation (periapical)
Gupta and Agarwal [20]	Soft tissue healing Pain assessment Consumption of analgesics Edematization Soft tissue healing Trismus	Clinical evaluation: (VAS, edematization, trismus) Radiographic evaluation: (periapical)
Alzahrani et al. [13]	Alveolar ridge width Bone regeneration	Clinical evaluation Radiographic evaluation: periapical
Srinivas et al. [14]	Alveolar bone height Bone density	Clinical evaluation Histological evaluation
Dutta et al. [27]	Soft tissue healing Bone regeneration	Clinical evaluation Radiographic evaluation: periapical
Niedzielska et al. [28]	Alveolar bone height Width Bone density	Clinical radiographic evaluation (CBCT)
Nemtoi et al. [19]	Bone regeneration Tooth movement	Clinical evaluation radiographic evaluation (CBCT)
Guzmán et al. [29]	Soft tissue healing Bone quality	Clinical evaluation Radiographic evaluation (panoramic X-ray)
Dimofte et al. [30]	Bone density	Clinical evaluation Radiographic evaluation (CBCT and panoramic X-ray)

Abbreviations: CBCT: cone-beam computed tomography; RCT: randomized controlled trial; VAS: visual analog scale.

**Table 5:** Data summary of the outcomes from the RCTs.

Author	PRF Result on Alveolar Ridge Preservation (Width, Length, Depth) and/or Bone Tissue Quality	Result of Physiological Healing and/or Biomaterials in Preservation of the Alveolar Ridge (Width, Length, Depth) and/or Quality of Bone Tissue	Statistical Significance Yes/No	Effect of Platelet Concentrate Reported in the Study
Clark et al. [26]	PRF: Ridge height: 1.8 ± 2.1 mm; Coronal width: 2.9 ± 1.7 mm Medial width: 1.8 ± 1.3 mm Apical width: 1.5 ± 1.6 mm Bone quality: 46 ± 18%	Blood clot: Ridge height: 3.8 ± 2.0 mm. Coronal width: 2.9 ± 1.7 mm Medial width: 1.8 ± 1.3 mm Apical width: 1.5 ± 1.6 mm Bone quality: 487 ± 48 mg/cm^3^ *. PRF + FDBA: Ridge height: 1.0 ± 2.3 mm. Coronal width: 1.9 ± 1.1 mm Median width: 1.7 ± 1.2 mm Apical width: 1.6 ± 1.5 mm Bone quality: 3 ± 3%. FDBA: Crest height: 2.2 ± 1.8 mm Coronal width: 2.5 ± 1.1 mm Medial width: 1.5 ± 1.2 mm Apical width: 1.2 ± 1.3 mm Bone quality: 29 ± 14%.	Height: Yes (*p* < 0.005) Width: No details Bone quality: No details	PRF produced more vital bone compared to FDBA, and also preserved the bone crest similar to FDBA and better than the blood clot; in relation to A-PRF + FDBA there is no statistical significance in bone formation.
Revathy et al. [1]	PRF: First month: 11.28650 UH third month: 17.08300 UH sixth month: 20.21800 UH	No details	First month: (*p* = 0.061) Third month: (*p* = 0.000, <1%) Sixth month: (*p* = 0.000, <1%)	PRF improves bone healing and bone formation compared to the control side, with significant bone gain at 1, 3, and 6 months after surgery.
Castro et al. [10]	PRF: Coronal width: −2.2 ± 0.9 mm Medial width: −1.6 ± 0.9 mm Apical width: −1.2 ± 0.8 mm Buccal wall height: 0.2 ± 1.1 mm P/L wall height: −1 ± 0.8 mm Bone quality: 54.5 ± 5.6%	L-PRF: Coronal width: −2 ± 1.0 mm; Medial width: −1.8 ± −1.7 mm Apical width: −1.2 ± 0.8 mm; Buccal wall height: 0.2 ± 1.2 mm P/L wall height: −1.1 ± 0.9 mm Bone quality: 47.7 ± 7.9%. Blood clot: Coronal width: −2.2 ± 1.0 mm Medial width: −1.7 ± 0.8 mm Apical width: −1.4 ± 0.8 mm; Apical width: −1.4 ± 0.8 mm. Buccal wall height: −0.2 ± 0.8 mm; P/L wall height: −0.2 ± 0.8 mm. P/L wall height: −1.0 ± 0.9 mm Bone quality: 34.7 ± 6.9%.	Width: No (*p* > 0.05) Buccal height: No (*p* = 0.3) P/L:(*p* = 0.8 Bone quality: L-PRF vs. PRF: No (*p* > 0.05); PRF vs. blood clot Yes (*p* < 0.05)	Horizontal and vertical changes at 1 mm below the alveolar ridge (vestibular and palatal) are similar in the three sites. Higher values were reported with L-PRF (85.2%) and PRF (83.8%) filling in relation to the control group (67.9%). Histological and imaging analysis showed bone neoformation for the PRF groups but not in the control group.
Suttapreyasri and Leepong [2]	PRF: 0–8 weeks Height M-D: 0.7 ± 1.33 mm. Width: No details Quality: No details	Blood clot: 0–8 weeks Height M-D: 1.23 ± 1.14 mm. Width: No details Quality: No details	Height M-D: No (*p* > 0.005) Width: No details Quality: No details	PRF can stimulate bone regeneration in situ without waiting for a normal body response, however, due to the minimal number of cytokines in PRF, the effect of bone regeneration is limited and cannot maintain the shape of the alveolar ridge post-exodontia, being statistically insignificant at 1, 2, 4, 6, and 8 weeks.
Hauser et al. [21]	PRF: 8 weeks Height: M: −1.2 ± 0.40 mm D: −0.76 ± 0.25 mm	Blood clot: 8-week height: M: −0.77 ± 0.17 mm. D: −2.07 ± 0.81. PRF-FLAP: 8-week height: M: −0.86 ± 0.34 D: −2.15 ± 1.05	Height M-D: Yes mesial wall in the blood clot group (*p* < 0.05)	Use of PRF to fill the alveolus without a flap following tooth extraction is associated with improved healing of alveolar bone tissue and preservation of ridge width and bone architecture.
Sharma et al. [22]	PRF: Bone quality: Immediate: 87.816 ± 33.318 16 weeks: 91,980 ± 33,728	Blood clot: Bone quality: Immediate: 85.378 ± 28.211 16 weeks: 88.689 ± 28.5847	Bone quality: No (*p* > 0.05)	Bone generation was not statistically significant at week 16 in relation to the control group; however, it accelerated the neoformation of bone tissue in the alveolus.
Kumar et al. [5]	PRF: 2 months: 0.11± 0.10 4 months: 0.16 ± 0.11 6 months: 0.16 ± 0.11	Blood clot: 2 months: 0.13 ± 0.12 4 months: 0.19 ± 0.13 6 months: 0.23 ± 0.12	Bone quality: No (*p* = 0.24)	Bone tissues show no significant difference in relation to the control group at 2, 4, and 6 months (*p*: 0.10).
Zhang et al. [3]	PRF: 3 months Buccal ridge: 1.6000 ± 1.46416 Lingual ridge: 1.0000 ± 0.70711 Width: 1.0500 ± 0.77862	Blood clot: 3 months Buccal crest: 2.8000 ± 1.81487 Lingual crest: 2.0500 ± 1.29180 Width: 2.0760 ± 1.67149	No statistical differences	Significantly greater bone neoformation in the PRF group (*p* < 0.001) No statistically significant differences in the mean value of vestibular alveolar ridge height, lingual/palatal alveolar ridge height, and alveolar ridge width). Advantageous PRF in alveolar ridge preservation.
Kapse et al. [17]	PRF: Lamina dura: 8 weeks: 1.23 ± 0.10 16 weeks: 1.80 ± 0.07 Bone density: 8 weeks: 1.23 ± 0.09 16 weeks: 1.83 ± 0.07 Trabecular pattern 8 weeks: 1.20 ± 0.11 16 weeks: 1.87 ± 0.06	Blood clot: Lamina dura: 8 weeks: 0.40 ± 0.009; 16 weeks: 0.90 ± 0.12; Bone density: 8 weeks: 0.27 ± 0.08 16 weeks: 0.63 ± 0.09 Trabecular pattern 8 weeks: 0.30 ± 0.09 16 weeks: 0.50 ± 0.09	Lamina dura—8 and 16 weeks: Yes (*p* < 0.001) Bone density—8 and 16 weeks: Yes (*p* < 0.001) Trabecular pattern —8 and 16 weeks: Yes (*p* < 0.001)	Regarding bone healing (lamina dura, bone density, and trabecular pattern) (*p* < 0.001) was higher at week 16 in relation to week 8 in sockets with PRF.
Gupta and Agarwal [20]	PRF: 1 month: 18.75% ± 5.12 3 months: 51.47% ± 3.93 6 months: 77.63% ± 6.97	Blood clot: 1 month: 13.58% ± 4.87 3 months: 47.58% ± 3.17 6 months: 70.54% ± 5.76	1 month: Yes (*p* = 0.0023) 3 months: Yes (*p*= 0.0014) 6 months: Yes (*p* = 0.0012)	Bone regeneration in sites with PRF at the first, third, and sixth months is statistically significant in relation to the control group (*p* < 0.005).
Alzahrani et al. [13]	PRF: Alveolar ridge width: 1 week: 11.70 ± 2.37 4 weeks: 11.33 ± 2.30 8 weeks: 10.97 ± 2.33 Bone fill: 1 week: 68.82 ± 1.07%. 4 weeks: 74.03 ± 1.22%. 8 weeks: 80.35 ± 2.61%	Blood clot: Alveolar ridge width: 1 week: 13.01 ± 3.00 mm 1 week: 13.01 ± 3.00 mm 4 weeks: 12.04 ± 2.50 mm. 8 weeks: 11.54 ± 2.42 mm. Bone filling: 1 week: 74.05 ± 1.66%. 4 weeks: 81.54 ± 3.33%. 8 weeks: 88.81 ± 1.53%.	1- 4 weeks: Yes (*p* = 0.012) 1- 8 weeks: Yes (*p* = 0.036) 4–8 weeks: No (*p* = 0.37)	Alveolar ridge width loss in the PRF group (−0.97 mm—8.58%) was significantly lower compared to the control group (−1.92—13.54%) at 4 and 8 weeks; PRF increases the efficiency of cell proliferation thus decreasing long-term bone loss.
Srinivas et al. [14]	PRF: Bone height: 24 h: 13.93 ± 3.56 mm 3 months: 12.28 ± 3.84 mm Bone density (alveolus): 24 h: 319.79 ± 95.472 3 months: 564.76 ± 94.856 Periapical region: 24 h: 530.39 ± 203.289 3 months: 748.02 ± 202.878	Blood clot: Bone height: 24 h: 14.68 ± 4.32 mm 3 months: 12.78 ± 3.82 mm Bone density (alveolus): 24 h: 194.82 ± 78.986 3 months: 295.87 ± 87.217 Periapical region: 24 h: 518.84 ± 266.518 3 months: 613.15 ± 237.926	24 h—3 months—Without PRF Alveolar height Yes (*p* < 0.001) Bone density: Yes (*p* < 0.003 Periapical region: Yes (*p* < 0.043) 24 h—3 months—With PRF Alveolar height: Yes (*p* <0.001) Bone density: Yes (*p* < 0.001) Periapical region: Yes (*p* < 0.05)	Improved bone density was reported.
Dutta et al. [27]	PRF: Bone quality: Hard laminin: 1 month: −0.6 ± 0.16, 2 months: 0.4 ± 0.16 6 months: 1.1 ± 0.10 Overall bone density: 1 month: −0.4 ± 0.16 2 months: 0.4 ± 0.16 6 months: 1.2 ± 0.13 Trabecular pattern: 1 month: −0.6 ± 0.16 2 months: 0.3 ± 0.15 6 months: 1.3 ± 0.15	Blood clot Bone quality: Hard lamella: 1 month: −1.9 ± 0.1 2 months: −1 ± 0.14 6 months: 0.1 ± 0.10 Overall bone density: 1 month: −1.9 ± 0.10 2 months: −1.3 ± 0.21 6 months: 0.1 ± 0.23 Trabecular pattern: 1 month: −1.9 ± 0.10 2 months: −1.3 ± 0.21 6 months: 0.1 ± 0.17	Statistical significance: Bone regeneration with PRF at 1, 2, and 6 months (*p* < 0.05) in relation to the control	Bone healing of the lamina dura.
Niedzielska et al. [28]	PRF: Width: 9.43 ± 1.74 mm Height: 1.49 ± 0.84 mm Bone quality: A: 308.16 ± 128.15	Blood clot: Width: 9.15 ± 1.51 Height: 1.85 ± 0.86 Bone quality: A: 279.40 ± 136.23	No statistical significance immediate post-exodontic: width and height of alveolar process. There is statistical significance 6 months post-exodontic: width and height of alveolar process (*p* = 0.0085)	Changes in the alveolar process. Changes in alveolar process height.
Nemtoi et al. [19]	PRF Height: Immediate: 5 mm 6 months: 1.9 mm Bone quality—14 weeks: D1 (1250 HU)	Blood Clot: Height: Immediate: 4.8 mm 6 months: 2.9 mm Bone quality—8 weeks: D1 (1250 HU)	There is statistical significance in bone regeneration	Changes in the alveolar process. Changes in alveolar process height.
Guzmán et al. [29]	PRF: 60 days: 163.86 UH	Blood Clot: 60 days: 159.31 UH	There is a statistically significant difference at 60 days (*p* < 0.015)	Bone healing.
Dimofte et al. [30]	PRF: Monoradicular teeth with PRF, density increased (*p* = 0.00484) compared to the control side. Pluriradicular teeth. Bone density increased in mesial (*p* < <0.001) and distal (*p* = 0.00304) roots for the mandible. The same results were obtained for the maxilla where PRF was used: mesiovestibular (*p* < 0.001) disto vestibular (*p* < 0.001) palatal (*p* < 0.001) roots. The ridge preservation (width, length, depth): no details	Blood Clot: No details	There is a statistically significant difference (0.000484)	Improved bone healing. Improved bone density.

* Abbreviations: FDBA: freeze-dried bone allograft; HU: Hounsfield units; PRF: platelet-rich fibrin

## Data Availability

No new data were created in this study. Data sharing is not applicable to this study.
